# Breast Cancer Survivors’ Perception on Health Promotion and Healthy Lifestyle: A Systematic Review and Qualitative Meta-Synthesis

**DOI:** 10.3390/ijerph22071131

**Published:** 2025-07-17

**Authors:** Luca Guardamagna, Orejeta Diamanti, Giovanna Artioli, Lorenzo Casole, Matteo Bernardi, Francesca Bonadies, Enrico Zennaro, Gloria Maria Modena, Tiziana Nania, Federica Dellafiore

**Affiliations:** 1 Department of Orthopedics and Traumatology, Istituto di Cura Città di Pavia, 27100 Pavia, Italy; luca.guardamagna@grupposandonato.it; 2 Healthcare Professions, Veneto Institute of Oncology IOV-IRCCS, 35128 Padua, Italy; orejeta.diamanti@iov.veneto.it (O.D.); matteo.bernardi@iov.veneto.it (M.B.); francesca.bonadies@iov.veneto.it (F.B.); enrico.zennaro@iov.veneto.it (E.Z.); 3 Department of Biomedicine and Prevention, University of Rome Tor Vergata, 00133 Rome, Italy; 4 Department of Medicine and Surgery, University of Parma, 43121 Parma, Italy; giovanna.artioli@unipr.it; 5 Integrated Home Care and Home Palliative Care Units, Fondazione Conte Franco Cella di Rivara Onlus, 27043 Broni, Italy; lorenzocasole90@gmail.com; 6 Welfare Services Area, IRCCS Istituto Clinico Humanitas, 20089 Rozzano, Italy; maria.modena@humanitas.it; 7 Training Office, IRCCS Policlinico San Donato, 20097 Milano, Italy; tiziana.nania@grupposandonato.it; 8 Department of Life Health Sciences and Health Professions, Link Campus University, 00165 Roma, Italy

**Keywords:** breast cancer survivors, healthy lifestyle, health promotion, recurrence, meta-synthesis

## Abstract

**Aims:** To systematically review and synthesize qualitative research exploring the Breast Cancer Survivors (BCSs)’ perception of health promotion interventions and informing strategies to mitigate recurrence risk within five years post-treatment and improve clinical outcomes. Specifically, this study addresses the question: “How do women diagnosed with breast cancer perceive health promotion interventions for recurrence prevention?” **Design**: A systematic review and qualitative meta-synthesis were performed. **Data Sources**: A systematic search of scientific databases (CINAHL, MEDLINE, and Scopus) was undertaken in November 2024. The reference list was cross-referenced and hand-searched to identify additional articles. **Review Methods**: Studies were included if they met the following criteria: they were primary qualitative studies focusing on BCSs within five years post-treatment, involving participants who had completed surgery, radiotherapy, or chemotherapy in the same time frame, as this period is critical for monitoring recurrence and implementing health promotion interventions. Only studies published in peer-reviewed journals and written in Italian, English, French, or Spanish were considered, provided that an abstract and the full text were available. Moreover, eligible studies had to be conducted in high-income or middle-income countries. Studies were excluded if they focused exclusively on advanced or metastatic breast cancer, if they involved mixed cancer populations without reporting separate data for BCSs, or if they were non-qualitative studies or gray literature. The review study protocol was registered in the PROSPERO database (CRD42024626033). **Results**: The literature search identified 490 records, 13 articles from databases, and 3 articles identified via other methods (web and citation searching) that met inclusion criteria. A narrative synthesis approach allowed the emerging five themes: (I) *Challenges*, (II) *Self-motivation and empowerment*, (III) *The relationships as a facilitator*, (IV) *Barriers to change*, and (V) *Proactive support strategies*. **Conclusions**: Addressing internal and external factors that influence health behaviors is essential to improve adherence, reduce recurrence risk, and enhance quality of life. Tailored interventions, social support, and healthcare engagement are crucial in this effort. **Impact**: Our meta-synthesis highlighted significant challenges as well as valuable resources for health promotion among BCSs, suggesting practical and tailored approaches to improving the adoption of healthy behaviors, supported by relationships and targeted support strategies.

## 1. Background

Breast Cancer (BC) is the most prevalent malignancy among women worldwide and remains the leading cause of cancer-related mortality [1]. In 2020, 2.3 million women were diagnosed with a BC [1], and it has been estimated that 3.2 million women will receive a BC diagnosis by 2050 [2]. BC is hugely devastating [3] and has a high and vital impact on quality of life, and both physical and psychological patient outcomes [4]. In particular, women facing BC are often exposed to anxiety and depression after diagnosis [5], and the stress of living with the fear of relapse is their biggest burden [6].

Recurrence, defined as the reappearance of malignancy following initial treatment, marks a deterioration in clinical prognosis and contributes to significant psychological distress among Breast Cancer Survivors (BCSs). According to the National Cancer Institute, a Sis an individual who has completed primary treatment and is living either with or without evidence of the disease. This broad definition includes both those in long-term remission and individuals managing ongoing challenges related to the disease. Recurrence is further defined as “cancer that has recurred (come back), usually after a period of time during which the cancer could not be detected” [7]. Recurrence rates remain clinically relevant, with a pooled recurrence proportion of 12.2% within 1–4 years post-diagnosis, increasing to 14.3% between 5 and 9 years, and reaching 23.3% after 10 or more years [8]. Given the rising global incidence of BC [1], and the increasing risk of recurrence over time—especially in certain subtypes and stages—the long-term risk represents a major public health concern.

Recent evidence suggests that health-promoting behaviors, such as regular physical activity, healthy nutrition, and weight management, play a significant role in reducing the risk of BC recurrence within the critical first five years following primary treatment and improving long-term survival [9,10,11]. This timeframe is critical, as it represents the follow-up period during which the risk of cancer recurrence is highest and health promotion interventions may have the greatest impact on clinical outcomes and quality of life. Although much of the current evidence derives from studies that include survivors beyond five years post-diagnosis, these findings highlight the critical importance of adopting healthy behaviors early in the survivorship trajectory, particularly within the first five years following primary treatment, when the risk of recurrence is generally higher. This underscores the need for targeted interventions that support behavior change during this pivotal window [9,10,11].

Despite this new evidence, BCSs often do not follow recommendations, and it is estimated that 70% of them annually do not adhere to a healthy lifestyle [12,13,14,15,16]. Specifically, only 32% adhere to physical activity during their follow-up [17], and only 14% of BCSs decide to follow the correct nutrition [18]. Major guidelines for breast cancer survivors, including the ACS and WCRF/AICR recommendations, highlight the importance of adopting healthy behaviors such as regular physical activity, weight management, and balanced diets to reduce the risk of recurrence and improve overall health during early survivorship [10,19]. However, these guidelines often lack detailed specifications on optimal intensity, duration, and composition of interventions tailored for survivors within the first five years, indicating a gap that future research and clinical practice should address.

Some authors identified different reasons that could hinder changes in BCS behavior and adhesion to a healthy lifestyle [12,16]. For example, BCSs prefer to spend time with their children, not give up their nutrition habits, not stop drinking alcohol or smoking tobacco, or renounce their engagements [20,21]. Additionally, a rich qualitative literature described the BCS perceptions of physical activity, nutrition, and weight management, allowing us to understand better the reasons why BCSs do not adhere to health promotion behaviors, the difficulties encountered, and the strategic role of nurses in sustaining and promoting a healthy lifestyle [21,22,23].

Therefore, this study aims to address this gap by systematically reviewing and synthesizing qualitative research on BCSs’ perceptions of health promotion interventions. This will inform tailored strategies to mitigate recurrence risk within five years after completion of primary treatment and improve clinical outcomes. Specifically, this study uses a systematic review and meta-synthesis approach to answer the research question: ‘How do women diagnosed with BC perceive health promotion interventions for recurrence prevention?’

## 2. Materials and Methods

### 2.1. Study Design

A systematic review and qualitative meta-synthesis were performed. Specifically, to keep the original meaning of individual studies, a new, cohesive interpretation of the results was generated through meta-synthesis, a method for integrating and presenting qualitative findings [24]. This process also contributes to advancing clinical practice, informing intervention design, and supporting theory development [25].

**Figure 1:**
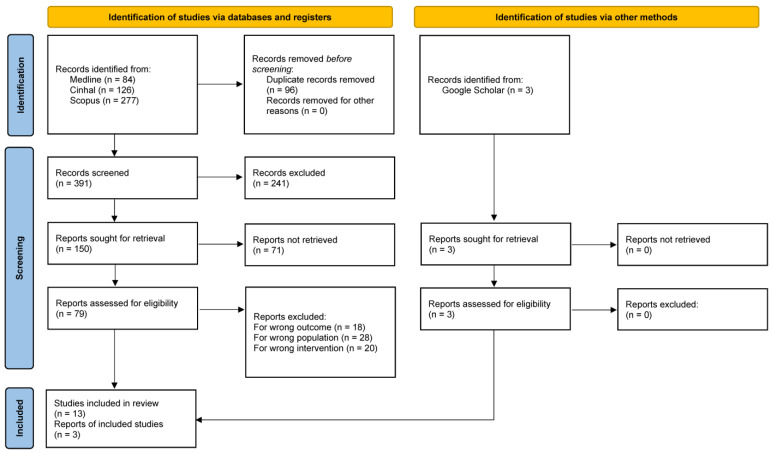
PRISMA flow chart [26].

The methodological criteria and PRISMA (Preferred Reporting Items for Systematic Reviews and Meta-Analyses) flowchart for systematic reviews (Figure 1) guided the identification of the primary literature to include in the meta-synthesis. It secured a detailed overview of the studies, while at the same time, it allows for an accurate analysis of every single research belonging to the sample [26]. Afterwards, the Joanna Briggs Institute (JBI) meta-aggregative approach in synthesizing qualitative evidence guided the included study’s synthesis [27]. The JBI approach to qualitative synthesis is designed to pragmatically identify evidence to guide clinical practice, employing a rigorous methodology [27]. This method followed a three-step process: firstly, generating statements that represent aggregated data by combining findings from qualitative studies; secondly, grouping findings into categories based on shared meanings; and thirdly, performing a meta-aggregation of the categories to create a cohesive synthesis of findings [27].

### 2.2. Protocol Registration

The review study protocol was registered with the International Prospective Register of Systematic Reviews (PROSPERO) of the National Institute of Health Research to straighten the study’s producibility, accountability, and transparency (CRD42024626033) [28].

### 2.3. Formulation of the Research Question

The search was constructed using the SPIDER (Sample, Phenomenon of Interest, Design, Evaluation, Research type) tool to define key elements of the review question better and to inform and standardize the search strategy. The SPIDER tool applied to the research question was as follows: Sample = adult women diagnosed with BC within the last five years, who completed primary treatment; Phenomenon of Interest = perception of health promotion measures, including nutrition, physical activity, and weight management; Design = phenomenology, ethnography, grounded theory or mixed-methods studies; Evaluation = effectiveness of health promotion interventions in reducing recurrence risk; Research type = literature review and synthesis [29].

### 2.4. Search Strategy

Tailored search strings were developed, incorporating specific keywords and Medical Subject Headings (MeSH Terms) such as Breast Cancer Survivors, Neoplasms, Meta-synthesis, Qualitative research, Experience, and point of view, with their variants. Boolean operators (AND, OR) were utilized to refine the search, and search strings were to adapt to each database’s requirements, ensuring efficient literature retrieval. Two independent reviewers (LG; FD) systematically searched three databases that provided broad coverage in nursing and medical domains: CINAHL, MEDLINE, and Scopus. MeSH and free-text words were listed to construct the search strategy, which was explicitly adapted to the syntax of each database as appropriate (Table 1). The reference list was cross-referenced and hand-searched to identify additional articles [30]. Theses, dissertations, abstracts in proceedings, systematic reviews, and other unpublished papers were excluded, as they are not subjected to a peer review. The review included articles published from January 2015 until November 2024.

### 2.5. Eligibility Criteria

SPIDER framework guided article selection [29]. The inclusion criteria were (a) articles (published and in press) consisting of primary studies focused on BCSs within the follow-up period of up to five years after the completion of primary treatment; (b) participants—defined as breast cancer survivors—who had completed surgical, radiotherapy, or chemotherapy treatments; (c) primary qualitative research, utilizing phenomenology, ethnography, grounded theory, or mixed methods (d) articles containing abstracts, (e) published in the Italian, English, French and Spanish language in peer-reviewed journals, and (f) full-text availability. Language restrictions were set to minimize any potential misinterpretation of the results.

Specifically, in this systematic review, we aimed to include studies investigating health promotion behaviors among breast cancer survivors (BCSs) across a broad survivorship continuum. We considered studies enrolling BCSs at various time points post-diagnosis, ranging from early survivorship (within 5 years of diagnosis or remission) to long-term survivorship (beyond 5 years). This approach was chosen to capture the dynamic nature of survivors’ motivations, barriers, and adherence patterns, which may change over time. When the exact duration since diagnosis or remission was not explicitly reported, studies were included if they otherwise met the inclusion criteria regarding population and outcome measures relevant to health promotion behaviors. Several included studies involved long-term survivors, with remission durations extending up to approximately 10 years or more. We intentionally retained these studies to provide a comprehensive and nuanced synthesis that reflects the heterogeneity of the BCS population and the evolving challenges in maintaining health-promoting behaviors throughout survivorship.

### 2.6. Data Selection and Extraction

Firstly, to support the screening organization and management, all records identified by databases were imported into Rayyan-Intelligent Systematic Review-software [31] (Version 7). The Rayyan^®^ platform is designed to optimize and streamline workflows for systematic reviews [31]. The process involved both automated and manual methods within Rayyan to remove duplicates, ensuring a thorough elimination of redundant records. After that, two authors independently (LG and LC) conducted a preliminary screening of the literature search by reviewing the titles and abstracts, followed by an in-depth examination of full texts. The assessment was conducted independently and anonymously by two reviewers to determine the relevance of the studies to the predefined research questions. Any conflicts were resolved through consultation with a third author (OD). The authors debated the reasons for inclusion or exclusion and disagreements to ensure reliability and consistency. The eligible articles selected during the title and abstract screening phase were then individually reviewed in full by the two authors [32]. Disagreements between the two authors were resolved during this phase through discussion with the goal of reaching a consensus. The structured approach guaranteed the selection of studies for review to be meticulous and unbiased.

### 2.7. The Methodological Quality of Studies

The JBI Critical Appraisal Checklist for Qualitative Research was used to assess the methodological quality of the studies [33]. The 10 items were evaluated using the following criteria: “*yes*,” “*no*,” “*unclear*,” and “*not applicable*.” The criteria evaluate the alignment between the study’s theoretical perspective, research methodology, data collection, analysis, and interpretation, while also ensuring that the researcher’s influence, ethical considerations, and participants’ voices are appropriately acknowledged and represented [33]. The tools were widely recognized for evaluating various research designs and allowed for a structured evaluation of study quality and relevance. Specifically, the evaluation results were determined based on the number of items meeting the standard requirements. Studies were classified as weak if the number of “yes” responses was ≤6, medium if between 7 and 8, and high if between 9 and 10. Only studies with at least a medium rating were included for data extraction and synthesis. Two reviewers (LG and FD) independently appraised the studies, with any disagreements resolved by a third reviewer (OD) to achieve consensus.

### 2.8. Data Analysis and Synthesis

Data from each study were systematically extracted into a standardized table summarizing key characteristics, including references, study purpose, design, and methodology with sample characteristics, key findings, and conclusions. The extracted data were synthesized to identify recurring themes and commonalities across the selected studies, with a thematic analysis conducted to derive meaningful insights. Data were meticulously extracted for qualitative studies to include codes, categories, and interpretations from the original study authors. These extracted data were abstracted into codes and subsequently consolidated into overarching themes or delineated into sub-themes where analytically appropriate. A synthesis of BCSs’ perceptions and the impact of health promotion interventions on recurrence prevention was encapsulated by the resulting themes.

## 3. Results

### 3.1. Study Selection

According to the PRISMA framework [26] that ensured a systematic, transparent, and comprehensive approach to identifying and analyzing the relevant literature, Figure 1 represents the screening methodology process for the identified studies. The literature search identified 490 records: 487 articles have been retrieved from databases CINAHL, MEDLINE, and Scopus, while 3 articles were identified through websites and citation searching. Before the screening started, 96 duplicate records were removed. Then, 394 records were screened based on title and abstract. Accordingly, 241 records were excluded because their content did not meet the inclusion criteria: they were not based on research methodology or not related to the research question. Then, of the 153 included records (150 from databases and 3 from other research), 71 were excluded because they were not retrieved (abstract or conference presentation). So, 82 full-text articles were screened for eligibility (79 from databases and 3 from other research), and 6 were excluded for the following reasons: 18 reported other outcomes, 28 described other populations, and 20 described other interventions. No study was excluded based on the quality critical appraisal tool. Finally, 13 articles from databases and 3 articles identified via other methods (web and citation searching) were included in the last PRISMA phase (Figure 1). The 16 included articles were analyzed using a narrative synthesis approach [34].

Table 2 shows the key characteristics of each included studies, such as aim, design, and methodology with sample characteristics, results, and conclusions, and Table 3 focuses on the description of various study qualitative design and sample features included.

### 3.2. Characteristics of the Included Studies

As represented in Table 3, the studies were conducted worldwide. All of them employed a qualitative design, emphasizing multiple specific approaches to explore BCSs’ nuanced experiences. Some studies used interpretive frameworks [10,35,36,37,38,39,40,41,44,45,46,47]; two studies adopted inductive and reflexive methods to identify themes from participant narratives [43,49], while Mehrabi et al. (2016) used a phenomenological design [50]. The total number of BCS patients enrolled in the 16 studies included was 230. The reported age range of participants spans from 18 to 72 years, reflecting the inclusion of both younger and older BCSs (Table 3).

Semi-structured interviews were the primary data collection method, valued for their flexibility in addressing specific topics while encouraging participant insights [35,36,37,38,39,40,41,43,44,45,46,47,49,50]. Instead, two studies used focus groups to provide collective insights, though influenced by group dynamics [42,48]. 

With regard to the methodological quality of included studies, most studies demonstrated strong methodological integrity and achieved rich data saturation, further supporting the reliability of the findings. Specifically, as showed in Table 2, the JBI Critical Appraisal Checklist for Qualitative Research of included studies was high, having a number of “yes” responses between 9 and 10 [33] Table 4.

The sample’s full text led to the identification of five main themes: (I) Challenges, (II) Self-motivation and Empowerment, (III) Relationships as a Facilitator, (IV) Barriers to Change, and (V) Proactive Support Strategies.

#### 3.2.1. Theme I: Challenges

The first theme emphasizes BCSs’ challenges in their commitment to health promotion. Generally, BCSs were determined to adopt behaviors that might mitigate recurrence risk, such as adhering to regular and intense physical activity programs, and to learn exercises suitable for their condition, start eating properly at all meals, and understand the correct foods to eat [49]. But this should not be overlooked. Some survivors faced conflicts between maintaining dietary discipline and the social aspects of eating, as one participant remarked “*I don’t want to be like ‘No, I actually can’t eat because of dietary restrictions that I put on myself because I’m fearful that my cancer’s going to come back’*” [36]. Structural constraints, such as time conflicts and familial responsibilities, further compounded the issue, with a young mother stating “*I probably wouldn’t participate [in a physical activity program] because I don’t want to miss out on [family] time*” [36], underlining the criticality of giving priorities: spend time with your children or join programs. Indeed, most BCSs do not take time away from their children to train, but at the same time, they do not want to give up their health either: “*Me being a young mom, I’m not [physically active]. I don’t want to miss out. My son goes to school, and if it [a physical activity program] is any time during his time at home, I probably won’t participate because I don’t want to miss out on that time*” [36]. The physical exhaustion associated with survivorship was a recurring theme, as one participant explained “*Usually I’m okay, but some days, I feel like I’d faint… When exhausted, you have to sleep… Can’t go out, it’s just like ‘playing dead*” [48]. Furthermore, the psychological burden of cancer survivorship was widespread, with one participant stating “*Every day is a battle with the fear of recurrence. Even if I feel fine, the thought is always there*” [50]. Physical and emotional struggles, however, remained significant. The challenges that BCSs faced were related to their self-image and the long-term effects of treatment. One survivor described the impact of chemotherapy on her body, saying “*I felt more pain in my body after chemotherapy… it caused me worry that if the pains and disabilities continue forever*” [50]. Others struggled with body image issues, as one survivor shared “*I took a shower without turning the light on for 2 months. I didn’t want to see it [my body]*” [42]. The BCSs’ efforts to embrace health-promoting behaviors are multifaceted, which are highlighted by these challenges.

#### 3.2.2. Theme II: Self-Motivation and Empowerment

Despite significant challenges experienced, many women were determined to adopt behaviors that might mitigate recurrence risk. In this process, motivation is a positive or negative determinant factor. One BCS shared the importance of physical activity: “*I’m finding it hard to stay active as my motivation is way low… Then days pass until I say I have to get to it again*” [41]. On the contrary, a 62-year-old BCS in Fang and Lee’s (2016) study asserts “*Now I want to change and think of [eating] all kinds of foods. […] Ah, we are scared of relapse, so we try to eat some alkaline items*” [37]. Another survivor also reflected on the impact of dietary changes, stressing the importance of self-motivation: “*In the past, I wouldn’t be able to get through a 10-hour day, but now I can. I’m tired by the end of the day, but not mentally tired, so eating the right foods is helping my mental status*” [38].

Regarding physical activity, another BCS states “Because of [BC], we must exercise; otherwise, you just lay there or sit here, which cannot be good. I have to get out and exercise or occasionally clean the house” [37]. Physical exercise has an essential mental benefit, too: “You really need to focus on mental benefits of exercise for BC, and control is a big issue too. You are dealing with not being in control, but exercise is something you can do” [40]. Structured programs recognized strong motivation: “Knowing that I was part of a structured program kept me going. The encouragement I received made me want to do better for myself” [39].

Additionally, making healthier choices improved their sense of empowerment: “*Knowing that I could influence my recovery with better choices motivated me to eat more healthily*” [50]. Similarly, the diagnosis itself served as a pivotal moment for self-awareness, with a participant noting “*It [cancer diagnosis] made me very conscious of the control I had of my health after the treatment had finished*” [43].

#### 3.2.3. Theme III: Relationships as a Facilitator

The third theme defines the importance of relationships: social support and empathetic healthcare providers were prominently highlighted as BCSs’ survivorship facilitators. Participants affirm that having trusted people to talk to is essential to improve their response to diagnosis treatments and to increase adherence to health promotion. Specifically, social support and peer connections offered emotional solace and practical advice, as one survivor noted “*You met a lot of other people [at a support group] who were able to tell you things that they found useful or didn’t find useful*” [43].

Innovative support mechanisms, like shared cooking classes or group exercise programs tailored to survivors, have been proposed as effective ways to promote a sense of community and support [36]. Therefore, one participant shared that those social interactions also mitigated feelings of isolation: “*My neighbours are very kind and comforting. They always ask me to go outside, and they chat with me*” [35].

On the other hand, self-care can be hindered by a support network that lacks companionship for activities such as physical exercise. As one participant noted, “*Not having someone to go with, that prevents me from doing it [physical activity] sometimes*” [41]. Empathetic healthcare providers were also essential, as their attention to BCS patients provided emotional security. One participant expressed “*If the doctor cares for me, I feel okay; thus, I am not afraid when the doctor is present*” [37]. Furthermore, another BCS affirmed “*Once, after I had a very long day, I grabbed a nurse and just started to express all the things that made my life so hard, and at some point, I found this nurse’s tears dropping on me! I was so touched and felt grateful to her, a huge help it was*” [48].

#### 3.2.4. Theme IV: Barriers to Change

The results of the included studies frequently identified several barriers to adopting health-promoting behaviors. Firstly, the misinformation and lack of guidance are often experienced by BCS: “*In this regard, we did not receive sufficient training. I do not know what food is good for me*” [44]. Other patients expressed dissatisfaction with structured dietary programs, highlighting personal preferences as a challenge: “*Some of the meals didn’t taste great, and it was hard to eat things that I wasn’t used to. I wished the meals were more tailored to my preferences*” [39]. Additionally, physical exercise can be hindered by physical and logistical challenges, making it necessary to use tailored programs. One participant emphasized the importance of exercises suited to their physical limitations: “*It would be excellent to have a program that considers that and helps with stretching and such*” [48]. Another prevalent theme was the absence of structured support during and after treatment: “*I didn’t get any of this information through treatment*” [40]. For some, the absence of post-treatment guidance felt isolated: “*It was like being pushed into the sea from shore on a boat with no oars … what now? What do I do? … I’ve had to do all this myself, and I don’t think that’s right*” [43]. Fatigue and emotional exhaustion became significant barriers as well. One participant noted, “*I felt exhausted… I had no energy, so I went to a well-being centre far away from my house*” [48]. Emotional challenges, such as body image struggles and strained relationships, appear to worsen the situation. Women shared “*When I look in the mirror, I don’t see the breast I should have had. I’ve accepted it. My husband has also accepted it. But it is still difficult*” [46]. Finally, financial hardships also emerged as a barrier to accessing treatments or therapies that could aid in health promotion, as one participant lamented “*My pension is not sufficient to pay for my medicine and treatment*” [44].

#### 3.2.5. Theme V: Proactive Support Strategies

Fortunately, women’s experiences identified many proactive support strategies for adopting a healthy lifestyle, especially to compensate for support gaps, emphasizing the importance of tailored interventions to promote sustainable health behaviors. BCSs can benefit from joining the health promotion program. It increases their physical performance and improves their body’s emotional and physical condition; it positively impacts life despite the diagnosis of BC. This is possible thanks to the information provided by dieticians and trainers regarding nutrition and physical activity that they must carry on having a healthy lifestyle [39]. “*We need regular exercise*” [35] and “*I [need] sports. I try eating healthy food*” [47]. Another participant in Kim’s (2020) study shared “*[…] we do need to raise the arm and use it for exercising. It would be excellent to have a program that considers that and helps with stretching. […] I think we need a light-intensity program*” [42]. Women’s organizational skills could help them maintain a healthy lifestyle after surgery, as they must manage work, medical visits, and other events, as well as physical activity and food preparation. In addition, they must address symptoms that could decrease the body’s response: fatigue, joint pain and swelling, dizziness, nausea, tingling, and asthenia are the most common. The changes associated with diagnosis and treatment cause increased mental strain that negatively impacts BCSs, sometimes causing difficulties in planning their diet. An alternative solution to having a healthy lifestyle emerged from the studies include asking family members for help and preparing the daily routine by scheduling all the commitments so that BCSs can have time for self-care. Alternative strategies, such as consuming anti-cancer foods, were mentioned by some patients to overcome the perception of inadequate medical support: *Thus, I have started consuming more fruits high in fibre and vitamins such as apples; I […] learned that fruits with fibre and vitamins could help fight free radicals, boost the immunity of the body and kill cancer cells*” [45].

## 4. Discussion

Adopting a healthy lifestyle is a key strategy for reducing the risk of cancer recurrence in BCSs, but encouraging behavioral change remains a significant challenge for healthcare systems and providers. Understanding BCSs’ perspectives and perceptions regarding their adherence to health-promoting behaviors is crucial. Our meta-synthesis offers a comprehensive overview, helping clinicians and researchers navigate the diverse findings of primary studies and provide adequate support. The results highlight the complex factors influencing BCSs’ adherence to healthy behaviors. The five themes identified—Challenges, Self-Motivation and Empowerment, Relationships as Facilitators, Barriers to Change, and Proactive Support Strategies—reveal the difficulties and potential solutions for improving adherence to health-promoting behaviors. Despite recognizing the benefits of physical activity and dietary changes, BCSs often face emotional distress, physical exhaustion, and structural barriers, which hinder their ability to maintain these behaviors. Nevertheless, self-motivation, supportive relationships, and structured intervention programs are essential in overcoming these obstacles.

BCSs face significant challenges in balancing health maintenance with family responsibilities, particularly motherhood. The demands of caring for children and family members often leave little time or energy for self-care, making it difficult for many BCSs to prioritize their own health needs, despite understanding the importance of healthy behaviors in preventing recurrence. This internal conflict, often influenced by cultural values that emphasize self-sacrifice, can create substantial barriers to adopting health-promoting behaviors [51]. Reframing self-care as an act that benefits both personal health and family well-being, along with fostering self-compassion and emotional resilience, may offer critical support in overcoming these barriers, allowing BCSs to maintain healthy behaviors even during times of high caregiving demands [52].

The unpredictable nature of BC recurrence presents a significant challenge not only for clinical management but also for the psychological well-being of survivors [53]. This uncertainty often generates a constant state of fear and vigilance, which can undermine survivors’ confidence in their ability to maintain health-promoting behaviors over time. Survivors may struggle with feelings of helplessness and a lack of control, which can erode motivation and lead to disengagement from healthy routines, especially when the perceived risk of recurrence feels beyond their personal influence **[53]**. Therefore, this inherent unpredictability highlights the critical importance of developing tailored, long-term health promotion and preventive strategies that address each survivor’s unique medical risk profile, psychosocial context, and evolving life circumstances. Survivors need not only clinical follow-up but also sustained, personalized guidance that integrates emotional support, empowerment, and skills to adapt lifestyle behaviors over time, even when facing fluctuating risk perceptions or changing personal responsibilities. In this sense, shifting from a static model of survivorship care to a dynamic, individualized approach is essential. This includes providing survivors with tools to cope with uncertainty, strengthen self-efficacy, and maintain resilience. For example, psychoeducational programs that combine health literacy with stress management, mindfulness, or cognitive–behavioral strategies may help reduce fear of recurrence and reinforce motivation for healthy behaviors [52,54]. Regular, personalized risk communication and goal-setting sessions can further empower survivors to feel actively engaged in their own long-term prevention plan. Addressing both the biological and psychosocial dimensions of recurrence risk is therefore vital to empowering BCSs to sustain health-promoting behaviors, enhance their quality of life, and potentially reduce the likelihood of recurrence.

Self-motivation and empowerment are central to the adoption of healthy behaviors among BCSs. Despite physical and psychological challenges, many survivors find motivation, often driven by the fear of recurrence. However, motivation is complex, shaped by social support, emotional resilience, and personal agency [55]. Intrinsic motivation, particularly the desire to regain control over one’s health, is crucial for maintaining an improved diet and physical activity [56]. Empowerment reinforces this drive, with BCSs showing a more significant commitment to health-promoting behaviors when they believe their choices can influence recovery. The diagnostic moment, a turning point in their journey, increases health awareness and encourages proactive self-care [54]. Recent studies underscore the importance of emotional resilience and self-compassion in maintaining motivation, significantly when family responsibilities and fear of recurrence complicate adherence [54].

Having strong social relationships is crucial to promoting survival and adhering to healthy behaviors. Support networks reduce psychological distress and enhance quality of life, with peer support—especially from those with similar experiences—building resilience and hope [57]. Support groups provide essential spaces for emotional exchange and practical advice during and after treatment, which can significantly improve physical and mental well-being. Inadequate social support, however, can undermine self-care and adherence to health-promoting behaviors, which necessitates targeted interventions to strengthen these networks [58]. Even so, the difficulty of accessing these resources is still a significant obstacle, especially for those in rural or underserved areas [59]. Future research should focus on scalable interventions, such as digital platforms, to support survivors, especially in regions with limited access to in-person services [60].

Access to healthy food and physical activity resources plays a crucial role in the well-being of BCSs, influencing their ability to maintain a healthy lifestyle and mitigate long-term treatment effects. Women with higher socioeconomic status have been found to experience a lower risk of BC recurrence, fewer subsequent events, and a more favorable prognosis following recurrence over a 10-year period compared to those with lower socioeconomic status [61,62]. This association may be attributed to various factors, including BC risk, adherence to adjuvant treatments, and the management of recurrence, all of which may be influenced by socioeconomic factors [62,63].

Access to affordable, nutritious food—such as fruits and vegetables—is often limited in low-income or rural areas, where the cost of healthy food is a well-documented barrier to adequate consumption [64]. Similarly, the cost and accessibility of physical activity facilities, including gyms or recreational centers, can be a barrier to exercise [65]. In this context, knowledge about how to engage in appropriate physical activity at home becomes essential. However, studies indicate that many survivors lack guidance on how to safely and effectively adapt physical activity routines to the home setting. There is often insufficient information available to BCSs about exercises tailored to their specific needs, particularly in the absence of professional supervision [66]. As previously discussed, cultural and socioeconomic factors substantially influence lifestyle behaviors. These barriers should be addressed through tailored educational interventions, improved access to affordable resources, and the implementation of community-based programs that promote healthy eating and physical activity. Such strategies are crucial to improving the long-term health outcomes of BC survivors [67].

BCSs also face significant barriers to adopting health-promoting behaviors due to a lack of information and training on healthy habits, such as diet and exercise. Some authors note that many survivors are uncertain about beneficial lifestyle changes after treatment, which leads to confusion and frustration [68]. Personalized interventions are essential to bridge these gaps, ensuring survivors are motivated and equipped to engage in healthy activities [69]. Proactive support strategies, including health promotion programs and dietitians’ and fitness trainers’ guidance, can improve physical and emotional well-being [49]. Organizational skills and family involvement are also crucial for maintaining a healthy lifestyle. By scheduling self-care and seeking support from loved ones, survivors can better balance health maintenance with family and work responsibilities [42]. These findings emphasize the importance of planning, coordination, and personalized approaches to achieving and sustaining a health-oriented lifestyle [42,49].

The complexity of BC, including its biological subtypes, stages, and extent, deeply affects treatment options and their duration. Even patients with the same cancer type may respond differently to treatments, leading to varying survival outcomes and psychological well-being. Surgical choices—from partial breast conservation to bilateral mastectomy—impact postoperative recovery and follow-up care. Additionally, the use of radiotherapy and the type of medical treatments, such as hormonal, chemotherapy, or targeted therapies, influence patients’ follow-up experiences. These factors affect not only recurrence rates but also patients’ anxiety levels and the frequency of follow-up visits. Such clinical variations highlight the need for personalized care approaches to support survivors’ adherence to healthy behaviors and overall well-being.

### 4.1. Strengths and Weaknesses of the Study

This study’s main strength is its thematic synthesis of qualitative research, which gives a comprehensive understanding of the challenges and facilitators that influence the adoption of health behavior among BCSs. The research captures various perspectives and insights by synthesizing multiple studies. The inclusion of direct patient quotes further enhances the validity and relatability of the findings, providing an authentic representation of BCSs experiences, as noted by Onwuegbuzie and Leech [70]. However, some limitations should be considered. Self-reported data may introduce recall bias, leading to overestimating or underestimating adherence to health-promoting behaviors. Moreover, the review includes a limited number of studies from each country, often with small or localized samples, which may not fully capture the diverse cultural perspectives on BC, health promotion, and healthy lifestyles. Therefore, the findings should be interpreted with caution regarding their generalizability across different populations and cultural contexts. Cultural and socioeconomic factors also play a significant role in shaping survivors’ behaviors and may affect the generalizability of the results across diverse populations.

Moreover, some cultural and socioeconomic contexts play a critical role in shaping BCSs’ health behaviors, including the normativity and feasibility of physical activity, especially among women balancing family responsibilities. For instance, in some countries or less resourced areas, exercise may not be a culturally accepted or practical option for women with caregiving duties. Many included studies lacked detailed descriptions of participants’ cultural backgrounds or geographic contexts, limiting the representativeness of the findings. Therefore, caution is warranted when generalizing results, as these factors may significantly influence survivors’ ability and motivation to engage in health-promoting behaviors. Financial constraints, limited social support, and healthcare disparities may vary significantly depending on demographic characteristics, limiting the findings’ broader applicability [59]. Future quantitative research is needed to address these gaps and assess the prevalence and significance of the identified themes across different populations [42].

### 4.2. Clinical and Public Health Implications

This study’s findings have significant implications for clinical practice and public health. Healthcare providers should prioritize individualized counseling that addresses specific barriers and motivations for each survivor. As supported by Kim et al. (2020), tailoring interventions to the unique needs of everyone can significantly enhance adherence to health-promoting behaviors [42]. Programs incorporating peer support, such as group exercise or community-based dietary workshops, can help build resilience and foster a sense of community, which has been shown to improve adherence and overall well-being. Educational interventions are also crucial in dispelling myths and providing accurate information, empowering survivors to make informed decisions about their health [49]. Furthermore, fatigue management strategies, including customized exercise programs with adjustable intensity, should be incorporated into survivorship care plans to help manage BCSs’ physical challenges and improve recovery [43]. These findings suggest that a more holistic approach, addressing emotional and physical needs, is essential for improving health outcomes in BCS.

In addition to individualized counseling and health-promoting interventions, our findings emphasize the critical need for a comprehensive follow-up care approach that goes beyond periodic imaging and symptom education. Integrating routine psychosocial support, ongoing lifestyle guidance, and proactive monitoring for signs of recurrence can enhance early detection and empower BCSs to maintain healthy behaviors consistently. Such a holistic follow-up framework should involve multidisciplinary teams, including oncologists, surgeons, dietitians, and mental health professionals, to address the complex and evolving needs of survivors after primary treatment completion.

## 5. Conclusions

This study highlights the multifaceted nature of health behavior adoption among BCSs, identifying obstacles and potential solutions. While numerous barriers exist, leveraging self-motivation, structured programs, and social support can significantly enhance adherence to health-promoting behaviors. By addressing individual needs and incorporating tailored interventions, healthcare providers and policymakers can play a pivotal role in improving survivorship outcomes and reducing the risk of recurrence among BCS. There is an urgent need to implement comprehensive preventive strategies that target both the primary onset of cancer and the risk of disease recurrence, reflecting a critical public health priority.

Future research should focus on developing and testing intervention models tailored to the unique needs of BCSs. Longitudinal studies are needed to evaluate support strategies’ long-term adherence and effectiveness. Additionally, exploring the impact of digital health tools, such as mobile applications for activity tracking and virtual support groups, could offer innovative solutions to improve lifestyle adherence among survivors. Understanding the intersectionality of factors such as socioeconomic status, cultural beliefs, and access to healthcare can further refine interventions to ensure their inclusivity and efficacy.

## Figures and Tables

**Table 1:** Search strings.

Databases	Search Strings	Data	Number of Results
**MEDLINE**	((((breast[Title/Abstract]) OR (chest[Title/Abstract]) OR (mammary glands[Title/Abstract]) OR (bosom[Title/Abstract])) AND ((cancer[Title/Abstract]) OR (tumor[Title/Abstract]) OR (carcinoma[Title/Abstract]) OR (malignancy[Title/Abstract]) OR (tumefaction[Title/Abstract]) OR (neoplasia[Title/Abstract]) OR (neoplasm[Title/Abstract]) OR (malignancies[Title/Abstract]) OR (malignant neoplasms[Title/Abstract])) AND ((survivor[Title/Abstract]) OR (recurrence[Title/Abstract]) OR (relapse[Title/Abstract]) OR (repetition[Title/Abstract]) OR (long-term survivor[Title/Abstract]) OR (long term survivor[Title/Abstract]) OR (survivor, long term[Title/Abstract]) OR (survivor, long-term[Title/Abstract])) AND ((risk factor*[Title/Abstract]) OR (diet[Title/Abstract]) OR (nutrition[Title/Abstract]) OR (physical activity[Title/Abstract]) OR (exercises[Title/Abstract]) OR (activity, physical[Title/Abstract]) OR (activities, physical[Title/Abstract]) OR (physical activities[Title/Abstract]) OR (physical exercise*[Title/Abstract]) OR (aerobic exercise[Title/Abstract]) OR (exercise, aerobic[Title/Abstract]) OR (exercise training*[Title/Abstract]) OR (obesity[Title/Abstract]) OR (overweight[Title/Abstract]) OR (lifestyle[Title/Abstract]) OR (quality of life[Title/Abstract]) OR (habits[Title/Abstract]) OR (weight management[Title/Abstract]) OR (weight loss[Title/Abstract]) OR (behaviour*[Title/Abstract]))) AND ((qualitative research[MeSH Terms]) OR (interviews as topic[MeSH Terms]) OR (focus group[MeSH Terms]))))	29 November 2024	n = 84
**CINHAL**	AB (breast OR bosom OR mammary glands) AND AB (cancer OR tumor OR neoplasia OR neoplasm OR carcinoma OR tumefaction OR malignancies) AND AB (survivor* OR recurrence* OR survivorship) AND AB (lifestyle OR diet OR (risk AND factor*) OR nutrition OR physical AND activity OR exercise OR overweight OR obesity OR (weight AND management) OR (weight AND loss)) AND AB (qualitative)	29 November 2024	n = 126
**Scopus**	TITLE-ABS-KEY (breast OR bosom OR (mammary AND glands)) AND TITLE-ABS-KEY (cancer OR tumor OR neoplasia OR neoplasm OR carcinoma OR tumefaction OR malignancies) AND TITLE-ABS-KEY (survivor* OR recurrence* OR survivorship) AND TITLE-ABS-KEY (lifestyle OR diet OR (risk AND factor*) OR nutrition OR physical AND activity OR exercise OR overweight OR obesity OR (weight AND management) OR (weight AND loss)) AND TITLE-ABS-KEY (qualitative)	29 November 2024	n = 277

**Table 2:** Key characteristics of included studies.

Reference	Aim	Method/Study Design	Results	Conclusions
Cheng et al., 2017 [35]	To explore the self-management experiences of Chinese BCSs.	Qualitative study utilizing secondary analysis of interview data; 19 participants with diagnosis in the last 5 years.	Identified themes include managing health and well-being, managing emotions, managing roles and relationships, and cultural influences on self-management.	Chinese BCSs actively engage in self-management practices influenced by cultural beliefs, highlighting the need for tailored interventions.
Milosevic et al., 2020 [36]	To investigate the attitudes and behaviors of young breast cancer survivors concerning physical activity, nutrition, and weight management.	Qualitative study using semi-structured interviews with 12 young BCSs.	Recognized conflicts between awareness of health benefits and the practical obstacles to achieving them; themes included sustaining a healthy lifestyle, navigating social expectations, and balancing personal and medical priorities.	Young BCSs face unique challenges in maintaining health-promoting behaviors, underscoring the need for tailored interventions addressing individual and contextual barriers.
Fang and Lee, 2016 [37]	To explore the experiences and needs of women BCSs, with a focus on their transition to survivorship.	Qualitative study involving semi-structured interviews with 13 long-term BCSs.	Themes included fear of recurrence, promoting health, seeking a body image, expectations for patient–physician relationships, and positive thinking.	Women transitioning to survivorship face unique psychological and social challenges, emphasizing the need for supportive interventions and resources tailored to their long-term well-being.
Coro et al., 2020 [38]	To investigate BCSs’ views on how diet impacts cognitive function and how cancer-related cognitive changes affect their dietary choices.	Qualitative study using semi-structured interviews with 15 cancer survivors (13 breast cancer, 2 colorectal cancer) analyzed through thematic analysis.	Identified themes include diet’s impact on cognition and cognition’s impact on diet.	Cancer survivors recognize a reciprocal relationship between diet and cognitive function, emphasizing the importance of personalized nutritional guidance and sustained support for long-term dietary adjustments.
Beckenstein et al., 2021 [39]	To explore the acceptability of a 22-week structured diet and exercise weight loss intervention among BCSs with overweight or obesity.	Qualitative study using semi-structured interviews with 17 BCS participants who completed the intervention; thematic analysis was conducted.	Four main themes emerged: “1. *facilitators of intervention adherence*, 2. *barriers of intervention adherence*, 3. *continuation of healthy habits post intervention*, and 4. *recommendations for* *intervention improvements*”.	Tailored interventions with group exercise, individualized meal provisioning, flexible self-monitoring methods, and tools for transitioning post-intervention are essential for improving adherence and outcomes among BCSs with overweight or obesity
Hirschey et al., 2017 [40]	To investigate the shared exercise outcome expectations among BCSs and examine how their cancer diagnosis and treatment shaped these expectations.	Mixed-method descriptive study using semi-structured interviews with 20 BCSs and a modified Outcome Expectations for Exercise (OEE) questionnaire. Data were analyzed using summative content analysis and descriptive statistics.	Three themes emerged: “1. *prevalence of common expectations*, 2. *pervasive impact of fatigue*, and 3. *a brighter future*”.	BCSs often have limited awareness of the benefits of exercise for managing long-term and late treatment effects, underscoring the need for targeted educational interventions to enhance understanding of exercise’s potential impact.
Brunet et al., 2013 [41]	To explore the barriers and motivators influencing physical activity participation among women treated for BC.	Qualitative study using semi-structured interviews with 9 BCSs (I to III cancer stage); thematic analysis was conducted.	Identified barriers include physical challenges, psychosocial factors, and environmental/organizational obstacles. Motivators included improving health, reducing fatigue, and enhancing quality of life.	Physical activity participation in BCSs is influenced by a complex interplay of barriers and motivators, highlighting the need for tailored strategies to support engagement.
S. Kim et al., 2020 [42]	To explore the experience of cancer-related fatigue (CRF), barriers to exercise, and facilitators of exercise adherence among BCSs.	Qualitative study using focus group (FG) interviews with 16 BCSs (6 participants with cancer stage I; 10 with cancer stage II) experiencing moderate-to-severe CRF. Four FG interviews were conducted. Thematic analysis was used to identify key themes.	Identified themes included the insidious nature of CRF, myths about exercise causing recurrence or lymphedema, multiple barriers, and facilitators.	CRF significantly impacts daily life and exercise adherence. Dispelling exercise-related myths and creating tailored, supportive exercise programs for BCSs are essential to promote adherence and manage fatigue effectively.
Deery et al., 2023 [43]	To explore BCSs’ attitudes towards health post-treatment, their awareness of co-morbidities, and access to support systems.	Qualitative study utilizing semi-structured interviews with 8 BCSs from Ireland and the UK, analyzed using thematic analysis.	Key themes included health and rehabilitation post-treatment and disparities in access to support services. BCSs highlighted the need for holistic, individualized care addressing diet, exercise, and stress management	Holistic rehabilitation and accessible support services are essential for improving cancer survivorship care. A cancer rehabilitation model, similar to cardiac rehabilitation, could provide comprehensive, lifelong support.
Khajoei et al., 2024 [44]	To explore the experiences and multidimensional needs of BCSs during survivorship.	Qualitative content analysis based on semi-structured in-depth interviews with 16 BCSs and four oncologists in Iran from April to July 2023. Data were analyzed inductively to extract central themes.	Identified themes included “1. *financial toxicity*, 2. *family support*, 3. *informational needs*, and 4. *psychological and physical issues*”	Identifying survivorship needs is critical for developing effective care plans. Financial, emotional, and informational support, along with addressing psychological and physical concerns, enhances BCSs’ quality of life.
Chumdaeng et al., 2020 [45]	To explore health behavior changes among Thai BCSs following treatment completion.	Qualitative descriptive study utilizing in-depth interviews with 15 BCSs (staged I to III), analyzed using content analysis.	Health behavior changes were categorized into three key areas: diet modifications to prevent recurrence, regular exercise to mitigate post-treatment complications, and strategies to reduce psychological distress through self-image improvement, relaxation, and spiritual practices	Adherence to health behavior changes is crucial for minimizing post-treatment complications and improving survivors’ quality of life. Tailored interventions by healthcare professionals can enhance these behaviors.
Drageset et al., 2016 [46]	To explore individual coping experiences and reflections of women following their first year after primary BC surgery.	Qualitative descriptive study based on individual interviews with 10 Norwegian women (staged I to II) who had undergone primary breast cancer surgery, analyzed using qualitative meaning condensation analysis.	Key themes included existential concerns, diverse emotional responses, active coping strategies, and a desire to return to normalcy. Participants often sought emotional and psychological balance through activities, relationships, and re-prioritizing life values.	Addressing the multidimensional coping strategies and varied needs of BCSs is essential. Tailored support from healthcare professionals can enhance BCSs’ adaptive coping and overall well-being.
Şengün et al., 2019 [47]	To explore Turkish BCSs’ experiences related to fear of recurrence (FOR).	Qualitative descriptive study using semi-structured interviews with 12 BCSs (staged I to III), analyzed using inductive content analysis.	Four themes emerged: “1. *Quality of fear*, 2. *Triggers*, 3. *Effects on life*, and 4. *Coping strategies*.”	Cultural and personal factors, such as fatalistic beliefs and familial roles, strongly influence FOR. Healthcare professionals should incorporate cultural sensitivity and provide supportive environments to address FOR effectively.
S. H. Kim et al., 2020 [48]	To explore the self-management needs of BCSs following treatment.	Qualitative FG interviews with 20 BCSs (3 participants with cancer stage I; 6 with cancer stage II; 11 participants with cancer stage III) in South Korea, analyzed thematically to identify key self-management needs. One FG was conducted.	Five themes emerged: “1. *Symptom management needs*; 2. *Emotional management needs*; 3. *Information acquisition needs*; 4. *Needs for relationships with healthcare providers*; 5. *Adaptation needs for adaptation*.”	Effective self-management interventions should address knowledge gaps, provide emotional and informational support, and promote tailored adaptations to improve survivorship outcomes.
Arem et al., 2024 [49]	To explore young adult cancer survivors’ perspectives on how cancer impacted various domains of their lives post-treatment.	Qualitative study using semi-structured interviews with 23 young adult cancer survivors (12 BCSs staged I to IV). Data analyzed using thematic analysis to identify core impacts on life domains.	Key themes included disruptions in relationships, challenges in education and career, financial burdens, and coping mechanisms such as hope, resilience, and redefining life priorities.	Comprehensive survivorship care should address financial, educational, and relational disruptions while promoting coping strategies like resilience and hope to improve quality of life.
Mehrabi et al., 2016 [50]	To explore the lived experiences and coping strategies of Iranian women confronting BC diagnosis and its implications.	A qualitative phenomenological study using semi-structured, in-depth interviews with 18 women diagnosed with BC. Data were analyzed thematically.	Two main themes emerged: “*emotional turbulence*,” including subthemes of uncertainty, fears, and worries, and “*threat control*,” including coping strategies like seeking support, adopting safe lifestyles, and relying on spirituality. Women experienced significant emotional distress but adopted various coping mechanisms.	Addressing the emotional and practical needs of BCSs is critical. Tailored interventions focusing on emotional support, lifestyle changes, and spiritual resilience can enhance coping and improve quality of life.

**Table 3:** Description of various study qualitative design and sample features included.

	Country	Study design	Intervention description	Age	Gender	*n*.
Cheng et al., 2017 [35]	China	Qualitative approach–Interpretative framework	In-depth interviews	41–65	Female	19
Milosevic et al., 2020 [36]	Canada	Qualitative approach–Interpretative framework	Semi-structured interviews	18–40	Female	12
Fang and Lee, 2016 [37]	Taiwan	Qualitative approach–Interpretative framework	In-depth interviews	48–72	Female	13
Coro et al., 2020 [38]	Australia	Contextualist approach-Interpretative framework	Semi-structured interviews	27–69	Female	13
Beckenstein et al., 2021 [39]	Canada	Qualitative approach–Interpretative framework	Semi-structured interviews	54–70	Female	17
Hirschey et al., 2017 [40]	USA	Qualitative approach–Interpretative framework	Semi-structured interviews	54–70	Female	20
Brunet et al., 2013 [41]	Canada	Qualitative approach–Interpretative framework	Semi-structured, in-depth interviews	/	Female	9
S. Kim et al., 2020 [42]	Korea	Descriptive approach-Interpretative framework	Focus group	20–69	Female	16
Deery et al., 2023 [43]	UK	Qualitative inductive and reflexive approach	Semi-structured interviews	45–64	Female	8
Khajoei et al., 2024 [44]	Iran	Descriptive approach-Interpretative framework	In-depth interviews	18–60	Female	16
Chumdaeng et al., 2020 [45]	Thailand	Descriptive approach-Interpretative frame-work	In-depth interviews	33–59	Female	15
Drageset et al., 2016 [46]	Norway	Descriptive approach-Interpretative frame-work	Interviews	48–68	Female	10
Şengün et al., 2019 [47]	Turkey	Descriptive approach-Interpretative frame-work	Semi-structured interviews	33–70	Female	12
S. H. Kim et al., 2020 [48]	South Korea	Qualitative approach–Interpretative framework	Focus group	41–64	Female	20
Arem et al., 2024 [49]	USA	Deductive–inductive approach	Semi-structured interviews	29–38	Female	12
Mehrabi et al., 2016 [50]	Iran	Phenomenological design	Semi-structured interviews	31–65	Female	18

**Table 4:** Included studies JBI Quality Appraisal.

	Authors	ITEM 1	ITEM 2	ITEM 3	ITEM 4	ITEM 5	ITEM 6	ITEM 7	ITEM 8	ITEM 9	ITEM 10	Rating
Cheng et al., 2017 [35]	LG	Yes	Yes	Yes	Yes	Yes	Yes	Yes	Yes	Yes	Yes	High
	FD	Yes	Yes	Yes	Yes	Yes	Yes	Yes	Yes	Yes	Yes	High
Milosevic et al., 2020 [36]	LG	Yes	Yes	Yes	Yes	Yes	Yes	Yes	Yes	Yes	Yes	High
	FD	Yes	Yes	Yes	Yes	Yes	Yes	Yes	Yes	Yes	Yes	High
Fang and Lee, 2016 [37]	LG	Yes	Yes	Yes	Yes	Yes	Yes	Yes	Yes	Yes	Yes	High
	FD	Yes	Yes	Yes	Yes	Yes	Yes	Yes	Yes	Yes	Yes	High
Coro et al., 2020 [38]	LG	Yes	Yes	Yes	Yes	Yes	Yes	Yes	Yes	Yes	Yes	High
	FD	Yes	Yes	Yes	Yes	Yes	Yes	Yes	Yes	Yes	Yes	High
Beckenstein et al., 2021 [39]	LG	Yes	Yes	Yes	Yes	Yes	Yes	Yes	No	Yes	Yes	High
	FD	Yes	Yes	Yes	Yes	Yes	Yes	Yes	No	Yes	Yes	High
Hirschey et al., 2017 [40]	LG	Yes	Yes	Yes	Yes	Yes	Yes	Yes	Yes	Yes	Yes	High
	FD	Yes	Yes	Yes	Yes	Yes	Yes	Yes	Yes	Yes	Yes	High
Brunet et al., 2013 [41]	LG	Yes	Yes	Yes	Yes	Yes	Yes	Yes	Yes	Yes	Yes	High
	FD	Yes	Yes	Yes	Yes	Yes	Yes	Yes	Yes	Yes	Yes	High
S. Kim et al., 2020 [42]	LG	Yes	Yes	Yes	Yes	Yes	Yes	Yes	Yes	Yes	Yes	High
	FD	Yes	Yes	Yes	Yes	Yes	Yes	Yes	Yes	Yes	Yes	High
Deery et al., 2023 [43]	LG	Yes	Yes	Yes	Yes	Yes	Yes	Yes	Yes	Yes	Yes	High
	FD	Yes	Yes	Yes	Yes	Yes	Yes	Yes	Yes	Yes	Yes	High
Khajoei et al., 2024 [44]	LG	Yes	Yes	Yes	Yes	Yes	Yes	Yes	Yes	Yes	Yes	High
	FD	Yes	Yes	Yes	Yes	Yes	Yes	Yes	Yes	Yes	Yes	High
Chumdaeng et al., 2020 [45]	LG	Yes	Yes	Yes	Yes	Yes	Yes	Yes	Yes	Yes	Yes	High
	FD	Yes	Yes	Yes	Yes	Yes	Yes	Yes	Yes	Yes	Yes	High
Drageset et al., 2016 [46]	LG	Yes	Yes	Yes	Yes	Yes	Yes	Yes	Yes	Yes	Yes	High
	FD	Yes	Yes	Yes	Yes	Yes	Yes	Yes	Yes	Yes	Yes	High
Şengün et al., 2019 [47]	LG	Yes	Yes	Yes	Yes	Yes	Yes	Yes	Yes	Yes	Yes	High
	FD	Yes	Yes	Yes	Yes	Yes	Yes	Yes	Yes	Yes	Yes	High
S. H. Kim et al., 2020 [48]	LG	Yes	Yes	Yes	Yes	Yes	Yes	Yes	Yes	Yes	Yes	High
	FD	Yes	Yes	Yes	Yes	Yes	Yes	Yes	Yes	Yes	Yes	High
Arem et al., 2024 [49]	LG	Yes	Yes	Yes	Yes	Yes	Yes	Yes	Yes	Yes	Yes	High
	FD	Yes	Yes	Yes	Yes	Yes	Yes	Yes	Yes	Yes	Yes	High
Mehrabi et al., 2016 [50]	LG	Yes	Yes	Yes	Yes	Yes	Yes	Yes	Yes	Yes	Yes	High
	FD	Yes	Yes	Yes	Yes	Yes	Yes	Yes	Yes	Yes	Yes	High

## Data Availability

Data sharing is not applicable.
